# Maternal Body Mass Index and Gestational Weight Gain and Their Association with Pregnancy Complications and Perinatal Conditions

**DOI:** 10.3390/ijerph16101751

**Published:** 2019-05-17

**Authors:** Martin Simko, Adrian Totka, Diana Vondrova, Martin Samohyl, Jana Jurkovicova, Michal Trnka, Anna Cibulkova, Juraj Stofko, Lubica Argalasova

**Affiliations:** 1 IInd Gynecology and Obstetrics Clinic, Faculty of Medicine, Comenius University, Bratislava 84199, Slovakia; cyklomartin@gmail.com (M.S.); md.adrian.t@gmail.com (A.T.); 2 Institute of Hygiene, Faculty of Medicine, Comenius University, Bratislava 84199, Slovakia; diana.vondrova@fmed.uniba.sk (D.V.); martin.samohyl@fmed.uniba.sk (M.S.); jana.jurkovicova@fmed.uniba.sk (J.J.); 3 Institute of Medical Physics, Biophysics, Informatics, and Telemedicine Faculty of Medicine, Comenius University, Bratislava 84199, Slovakia; michal.trnka@fmed.uniba.sk; 4 Institute of Foreign Languages, Faculty of Medicine, Comenius University, Bratislava 84199, Slovakia; anna.cibulkova@fmed.uniba.sk; 5 Institute of Physiotherapy, Balneology and Medical Rehabilitation, University of Ss. Cyril and Methodius in Trnava, 91701, Slovakia; juraj.stofko@gmail.com

**Keywords:** retrospective hospital-based study, overweight, obesity, pregnancy pathologies, caesarean section, weight gain

## Abstract

This study aimed to evaluate the impact of selected pregnancy pathologies statistically depending on overweight/obesity and excessive maternal weight gain during pregnancy on women who gave birth in the years 2013–2015 at the Second Department of Gynecology and Obstetrics at the University Hospital in Bratislava, Slovakia. In a retrospective study, we analyzed data gathered from the sample, which consisted of 7122 women. Our results suggest a statistically significant, higher risk for the groups of women with overweight and obesity and gestational hypertension (adjusted odds ratio (AOR) = 15.3; 95% CI 9.0−25.8 for obesity), preeclampsia (AOR = 3.4; 95% CI 1.9−6.0 for overweight and AOR = 13.2; 95% CI 7.7−22.5 for obesity), and gestational diabetes mellitus (AOR = 1.9; 95% CI 1.2−2.9 for overweight and AOR = 2.4; 95% CI 1.4−4.0 for obesity). A higher incidence of pregnancies terminated by cesarean section was observed in the group of obese women. Gestational weight gain above IOM (Institute of Medicine) recommendations was associated with a higher risk of pregnancy terminated by C-section (AOR = 1.2; 95% CI 1.0−1.3), gestational hypertension (AOR = 1.7; 95% CI 1.0−2.7), and infant macrosomia (AOR = 1.7; 95% CI 1.3−2.1). Overweight and obesity during pregnancy significantly contribute to the development of pregnancy pathologies and increased incidence of cesarean section. Systematic efforts to reduce weight before pregnancy through prepregnancy dietary counseling, regular physical activity, and healthy lifestyle should be the primary goal.

## 1. Introduction

The obesity epidemic has become a worldwide phenomenon not only from a medical point of view but also from a social one. The alarming increase in obesity worldwide has led the World Health Organization (WHO) to classify obesity as one of the most pressing global health issues of the 21st century [1]. Today, obesity is considered to be the most common metabolic disorder, which has become a global epidemic. Obesity is defined as excess body fat that is highly likely to lead to health deterioration, increased morbidity, and mortality. The high increase in the prevalence of obesity has also affected women of reproductive age. The most commonly used indicator of obesity is the body mass index (BMI). In 2009, based on the BMI, the Institute of Medicine (IOM) classified body weight into underweight (BMI < 18.5 kg/m^2^), normal weight (BMI = 18.5–24.9 kg/m^2^), overweight (BMI = 25.0–29.9 kg/m^2^), and obese (BMI ≥ 30 kg/m^2^). Based on the BMI, obesity has three levels: BMI 30.0–34.9 (class I), BMI 35.0–39.9 (class II), and BMI ≥ 40 (class III) or morbid obesity. Obesity in pregnancy is defined as BMI ≥ 30 kg/m^2^ at the first prenatal counseling visit. The IOM recommends a range of healthy weight gain amongst expectant mothers, for underweight (12.5–18.0 kg), normal weight (11.5–16.0 kg), overweight (7.0–11.5 kg), and obese (5.0–9.0 kg) [2]. Maternal obesity has become one of the most commonly occurring risk factors in obstetric practice [3,4,5].

Numerous experimental and epidemiological studies show that nutritional changes in prenatal and postnatal stages of life can have a significant impact on health and child development [6,7]. Professional scientific societies point to the fact that due to obesity in pregnancy, in the postnatal period and adulthood, there is a higher incidence of metabolic disorders, neurodevelopmental disorders, cancer, and adverse changes in the immunological functions of an individual [8,9,10,11]. In developed countries, most women of childbearing age are already overweight or obese before becoming pregnant. The number of obese pregnant women is rising, which poses a threat to the future health of children. The WHO reports that the prevalence of obesity during pregnancy ranges from 1.8% to 25.3% [1]. According to the European Perinatal Health Report [12], in most European countries, more than 30% of pregnant women are obese. The proportion of overweight or obese women ranges from 30% to 50%, with a prevalence of less than 30% in Croatia, Austria, and Slovenia and around 50% in the UK. Some EU countries, including Slovakia, do not systematically contribute to the database of the Euro-Peristat network on BMI data on maternal body weight. Therefore, in the literature, some European countries have no relevant data on maternal obesity.

Obesity in pregnancy is associated with an increase in pregnancy complications, such as the risk of miscarriage, fetal and congenital anomalies, thromboembolism, preeclampsia and gestational hypertension, fetal macrosomia, gestational diabetes mellitus, IUGR (intrauterine growth restriction), and stillbirth, as well as intrapartum and postpartum complications and neonatal mortality [13,14,15,16,17,18,19,20,21,22,23,24,25,26]. In connection with obesity, a higher number of cesarean sections [27,28] and a lower number of lactating women [29] are recorded, compared to women with a normal BMI. Obesity may be a risk factor for maternal mortality [30,31].

Gestational weight gain is also an important predictor of adverse maternal and neonatal health outcomes. Insufficient weight gain is associated with increased risks of preterm birth and delivery of a low-birth-weight infant, whereas excessive weight gain is associated with increased risks of gestational hypertension, preterm birth, delivery of a high-birth-weight infant, and cesarean delivery [32,33].

This study aimed to evaluate the impact of selected pregnancy pathologies statistically depending on overweight/obesity and excessive maternal weight gain during pregnancy on women who gave birth in 2013–2015 at the Second Department of Gynecology and Obstetrics at the University Hospital in Bratislava, Slovakia.

## 2. Materials and Methods

In our retrospective study, we analyzed a group of 7122 pregnant women during the period of 1 January 2013, to 31 December 2015. The study data were obtained from a computerized obstetrics database (Hospital Information System) and included demographic characteristics, medical and obstetric histories, and information on maternal and perinatal outcomes. We analyzed all singleton deliveries after 37 weeks of gestation, excluding pregnancies with chronic hypertension, fetal anomalies, and diabetes mellitus type 1 and 2. Women were categorized into four groups based on their prepregnancy BMI (underweight, normal weight, overweight, and obese) and three groups of gestational weight gain (GWG) relative to the IOM guidelines (inadequate, adequate, excessive) [2].

Prepregnancy weight was measured at the first antenatal visit during the first trimester of pregnancy; final pregnancy weight was measured at the last antenatal visit or the time of delivery. Body weight was assessed according to a standard protocol (barefoot, with light clothes on) using an electronic digital scale with the kilogram mode during each antenatal visit. BMIs were categorized according to the WHO’s classifications: Underweight (<18.5), normal weight (18.5–24.9), overweight (≥25.0), and obese (≥30).

Gestational weight gain was defined as the difference between the final weight, and the prepregnancy weight and was classified into three groups based on prepregnancy BMI and GWG relative to the IOM guidelines: (i) Weight gain below the guidelines, (ii) weight in the range, and (iii) weight gain above the guidelines.

We examined the following maternal outcomes: Preeclampsia, gestational hypertension, gestational diabetes mellitus (GDM), gestational hepatopathy, intrauterine growth restriction (IUGR), and cesarean delivery in relation to maternal advanced age (over 35). The neonatal outcomes examined were low birth weight (<2500 g) and macrosomia (>4000 g), which were defined according to the WHO’s birth weight classification [34]. The American College of Obstetricians and Gynecologists (ACOG) have adopted the definition of IUGR as an estimated fetal weight of less than 10th percentile [34].

Gestational hypertension was defined on the basis of a systolic pressure greater than or equal to 140 mm Hg or diastolic pressure greater than or equal to 90 mm Hg on two separate occasions 2–240 h apart after 20 weeks of gestation in the absence of proteinuria. Preeclampsia was defined as gestational hypertension with either proteinuria, which was defined as greater than or equal to 300 mg in a 24-h sample [35].

Gestational diabetes mellitus is defined as any glucose intolerance with the onset or first recognition during pregnancy. We used a 50 g oral glucose challenge test (OGCT) as a screening method for GDM at 24–28 weeks of gestation [36].

The outcomes for the second part of our analysis of gestational weight gain were gestational hypertension, preeclampsia, GDM, cesarean section delivery, and IUGR in relation to maternal advanced age (over 35), gestational age, and smoking.

Data obtained were statistically compared among particular groups of women. In each group, we analyzed and statistically evaluated the incidence of pathological conditions complicating the course of pregnancy. Indications for deliveries terminated by cesarean section were statistically evaluated and compared in particular statistical groups.

Regarding statistical analysis, the continuous variables were expressed as mean ± standard deviation. The comparison of the four prepregnancy BMI groups (underweight, normal weight, overweight, and obese) and three groups of gestational weight gain (GWG) relative to the IOM guidelines (inadequate, adequate, and excessive) was performed by ANOVA and multiple post-hoc group comparisons with Bonferroni adjustment. For categorical variables, the categorical Mantel–Haenszel analysis was used. A multiple logistic regression model controlling for maternal age, gestational age, gestational weight gain, and smoking was used to calculate the adjusted odds ratios (AOR) and 95% confidence intervals (CI) for adverse perinatal outcomes based on BMI and GWG. The reference categories were normal prepregnancy weight or adequate GWG relative to the IOM guidelines. Statistical significance was evaluated at the significance level *p* < 0.05. Statistical analysis was performed using SPSS software, Version 24. This study was approved by the University Hospital Ethics Committee No. EK/101/2018.

## 3. Results

Our group involved 7122 pregnant women, of whom 741 (10.4%) accounted for the category of women with maternal underweight, 5400 (76.0%) women with normal weight, 602 (8.5%) women with overweight, and 358 (5.0%) women with obesity. Because of missing data, 21 women were excluded from the total group, and 7101 women remained for the analysis.

Table 1 summarizes the results of the statistical analysis of the selected data of the women and the selected pathological conditions in pregnancy in the BMI categories, which indicated a statistical significance in the comparison of four BMI groups (underweight, normal weight, overweight, and obese). Obese women had a significantly higher prevalence of gestational hypertension (10.6%), preeclampsia (10.9%), and GDM (8.7%) than normal-weight and underweight women (*p* < 0.001). Pregnancy terminated by cesarean section was more often seen in the group of obese and overweight women (57%) than in the group of normal-weight women (34.7%; *p* < 0.001). The prevalence of IUGR was higher in the group of obese women (3.9%) than in the group of women with normal weight (1.2%; *p* < 0.001). In the group of underweight women, the prevalence of IUGR was 1.8%. In the group of obese mothers, the number of smokers was significantly higher (7.3%) than in the group with normal-weight mothers (1.6%; *p* < 0.001). The prevalence of infants with macrosomia was higher in overweight (9.3%) and obese women (9.6%) than in normal and underweight women (7.0%; *p* < 0.05).

Table 2 shows the results of the multivariable logistic regression analysis assessing the independent effect of overweight/obesity on selected maternal/fetal pathologies controlling for age, gestational age, gestational weight gain, and smoking. There was a positive association in the groups of women with overweight/obesity and pregnancy pathologies, such as gestational hypertension for obesity (AOR 15.3; 95% CI 9.0−25.8), preeclampsia for overweight (AOR 3.4; 95% CI 1.9−6.0) and obesity (AOR 13.2; 95% CI 7.7−22.5), gestational diabetes mellitus for overweight (AOR 1.9; 95% CI 1.2−2.9) and obesity (AOR 2.4; 95% CI 1.4−4.0), IUGR for obesity (AOR 3.7; 95% CI 1.8−7.8), and infant macrosomia for overweight (AOR 1.7; 95% CI 1.2−2.3) and obesity (AOR 1.8; 95% CI 1.2−2.7). In obese women, there was also a significantly higher risk of terminated pregnancy by C-section (AOR 2.1; 95% CI 1.7−2.8) and lower risk of a low-birth-weight infant (AOR 0.2; 95% CI 0.1−0.5). By contrast, underweight women had a significantly lower risk of caesarian delivery (AOR 0.8; 95% CI 0.6−0.9) and infant macrosomia (AOR 0.7; 95% CI 0.5−0.9).

**Figure 1:**
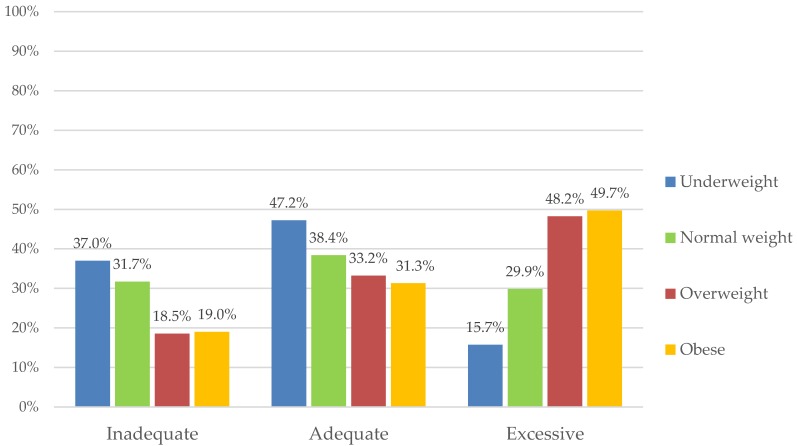
The proportion of maternal BMI categories in gestational weight gain groups (IOM recommendations). Legend: There is a significant difference between the distribution of overweight/obesity and underweight (*p* < 0.001) and normal weight (*p* < 0.001) in the inadequate gestational weight gain (GWG) group. In the adequate GWG group, there is a significant difference in the distribution of underweight compared to normal weight (*p* < 0.001), overweight (*p* < 0.001), and obesity (*p* < 0.001). In the excessive GWG group, significant differences are between the proportion of women in underweight and normal weight categories (*p* < 0.001) and in the overweight and obese compared to underweight (*p* < 0.001) and normal weight categories (*p* < 0.001).

Distribution of GWG according to BMI categories is illustrated in Figure 1. An adequate amount of weight gain was observed in 47.2% of the underweight women, 37% of whom were below the IOM range and only 15.7% had excessive weight gain during pregnancy. Inadequate weight gain was mostly seen in the underweight and normal BMI groups, whereas excessive GWG was observed in overweight and obese mothers. GWG above the recommended range was observed in 48.2% of the overweight mothers and 49.7% of the obese mothers. There were statistical differences between the distribution of underweight, normal weight, overweight, and obesity in each GWG group (*p* < 0.001).

A statistical analysis of selected pregnancy pathologies in women according to the GWG IOM recommendations is shown in Table 3. Women with excessive GWG had significantly higher prepregnancy BMI (22.74 ± 4.39 vs. 21.67 ± 3.64; *p* < 0.001), higher BMI before delivery (29.65 ± 4.01 vs. 26.53 ± 3.19; *p* < 0.001), longer gestation (39.31 ± 1.25 vs. 39.20 ± 1.33; *p* < 0.001), higher occurrence of cesarean delivery (38.5% vs. 34.0%; *p* < 0.05), higher occurrence of gestational hypertension (2.2% vs. 1.1%; *p* < 0.05), and higher incidence of infant macrosomia (10.3% vs. 6.1%; *p* < 0.001) compared to women with GWG in the normal range. Mothers with lower-than-recommended GWG had a higher incidence of IUGR (2.2% vs. 1.3%; *p* < 0.001) and a low-birth-weight infant (9.1% vs. 3.5%; *p* < 0.001) compared to those who gained the recommended amount of weight.

The relationships among GWG and selected maternal/fetal outcomes explored using multiple logistic regression are presented in Table 4. Excessive weight gain was significantly associated with increased risk of cesarean section (AOR 1.2 (95% CI 1.0−1.3)), gestational hypertension (AOR 1.7 (95% CI 1.0−2.7)), and infant macrosomia (AOR 1.7 (95% CI 1.3−2.0)) and lower risk of GDM (AOR 0.6 (95% CI 0.4−0.9)) compared to adequate weight gain during pregnancy. Women with weight gain lower than the IOM recommendations had lower risk of preeclampsia (AOR 0.5 (95% CI 0.3−0.9)), and were in a higher risk of delivering a low-birth-weight infant (AOR 2.0 (95% CI 1.5−2.7)) compared to mothers with adequate GWG.

## 4. Discussion

The prevalence of obesity in pregnant women worldwide ranges from 1.8% to 25.3% [1]. In our retrospective study, we had 602 (8.5%) overweight women and 358 (5.0%) obese women. A total of 7122 women who gave birth in those years in our hospital came from different regions in Slovakia and are working and living in the Bratislava agglomeration. In the observed period, although the women with overweight and obesity in our sample belonged to a lower limit of the obesity prevalence in pregnancy in the EU, based on our results, we can conclude that not only obesity with BMI ≥ 30.0 but also overweight with BMI between 25.0 and 29.9 is a high-risk factor for the occurrence of pathological conditions in pregnancy, such as preeclampsia, GDM, gestational hypertension, and IUGR [37,38]. Liu et al. [39] showed that compared to antenatal weight gain within the IOM recommendations, excessive weight gain increased the incidence of cesarean section, preeclampsia, and infant macrosomia and reduced the incidence of GDM, while inadequate antenatal weight gain increased the incidence of GDM and low birth weight. The results of our study are consistent with several publications that confirm that obesity is a significant risk factor contributing to a higher incidence of pregnancies terminated by cesarean section [40]. Within the given period, in our obese group, up to 57% of pregnancies were terminated by cesarean section; this figure significantly exceeds the national average of pregnancies terminated by cesarean section in Slovakia (31% in 2016) [41].

In our study, inadequate, adequate, and excessive gestational weight gain were observed: In the inadequate GWG group, 37.0% underweight, 31.7% normal weight, 18.5% overweight, and 19.0% obese; in the adequate GWG group, 47.2% underweight, 38.4% normal weight, 33.2% overweight, and 33.3% obese; in the excessive GWG group, 15.7% underweight, 29.9% normal weight, 48.2% overweight, and 49.7% obese. Gestational weight gain greatly differed per maternal prepregnancy BMI group and was gradually higher across higher BMI groups. It was concluded that obese women are more likely to exceed the GWG recommendations.

Both extremes, excessive or inadequate GWG, can lead to adverse pregnancy outcomes. According to several studies [42,43,44], women whose weight gain is outside the IOM-recommended ranges are also associated with a higher incidence of pregnancy complications compared to women with normal weight gain. In our analysis, we found that excessive weight gain is associated with hypertensive disorders in pregnancy, delivery of a macrosomic infant, and higher incidence of C-section, which corresponds to a number of published results [42,45,46]. In a study conducted in Ireland, maternal obesity and increased GWG were associated with an increased risk of cesarean section and preeclampsia [47]. Some studies have reported that weight gain above the recommendations is associated with an increased risk of delivering a macrosomic infant, whereas less-than-recommended weight gain is associated with an increased risk of delivering an infant with low birth weight, consistent with our results [48,49].

The rate of overweight and obesity is also increasing in the Australian obstetric population. Women who are overweight and obese have an increased risk of adverse pregnancy outcomes. In particular, obese women are at increased risk of developing gestational diabetes (relative risk (RR) 2.10 (95% CI 1.17, 3.79)), gestational hypertension, and preeclampsia (relative risk (RR) 2.99 (95% confidence intervals (CI) 1.88, 4.73)) [50].

In Nova Scotia, Canada, moderately obese women have an increased risk of developing gestational hypertension (AOR 2.38 (95% CI 2.24, 2.52)) and cesarean delivery (AOR 1.60 (95% CI 1.53, 1.67)). Severely obese women have an increased risk of developing gestational hypertension (AOR 3.00 (95% CI 2.49, 3.62)) and cesarean delivery (AOR 2.46 (95% CI 2.15, 2.81)) [51].

In our study, the increased adjusted risk of gestational hypertension, preeclampsia, GDM, cesarean delivery, IUGR, and macrosomia was higher in overweight and obese women, when adjusted for age, gestational age, GWG, and smoking.

The results of our study confirm associations between obesity/overweight and increased risks of pregnancy pathologies and adverse neonatal outcomes in a Slovakian cohort consistent with the other previously reported cohorts. Such data from Slovakia are not present in any accessible international database and peer-reviewed international journal as well. These facts make the study novel and valuable.

The strength of our study is its large sample of pregnant women obtained retrospectively from an electronic obstetric database of the Hospital Information System (HIS) of the University Hospital Bratislava, which includes the demographic, obstetric, and neonatal characteristics of hospitalized women. Its limitation is its restriction to the Bratislava agglomeration only, even though families from all regions of Slovakia come to the area for work. The other limitation is its short-time retrospective design (2013–2015) instead of a longitudinal study design. In the multivariate logistic regression analysis, we could adjust only for age, gestational age, and smoking; data on parity, socioeconomic, and marital status were not available.

The next issue is that we recorded maternal weight at the beginning of the first trimester of pregnancy, and it was considered as prepregnancy weight. This is commonly used in these types of studies. The prepregnancy weight was recorded only through self-report, and it could not correspond to the true prepregnancy weight.

We fully understand that studies on the impact of obesity and GWG require the use of more precise data collection methods and calculations of body weight and GWG. Researchers and clinicians should use uniform definitions and diagnostic criteria for maternal and neonatal outcomes to allow for a better comparison of maternal weight and GWG data and their influence on maternal and infant outcomes.

This study adjusted for a number of variables that were potential confounders, such as maternal age, gestational age, maternal BMI, and smoking. However, it remains possible that other unmeasured confounders are mediating the relationship between obesity/overweight and increased risks of pregnancy pathologies and adverse neonatal outcomes.

In spite of these limitations, our data provide information on maternal weight and GWG and their impact on pregnancy outcomes, and these findings could have important implications for the clinical practice.

## 5. Conclusions

In our study, we comprehensively analyzed the influence of prepregnancy BMI and GWG on perinatal outcomes. We confirmed that both prepregnancy BMI and GWG cause adverse perinatal outcomes.

Prepregnancy dietary counseling, regular physical activity, and a healthy lifestyle (uptake of exercise, dietary intake, avoiding smoking and alcohol) could help to reduce the incidence of gestational obesity and the incidence of perinatal complications as well. Systematic efforts to reduce weight before pregnancy and excessive GWG should be the primary goal. Achieving optimal weight for every pregnant woman is the basis for the development of a healthy population and an essential factor for the physiological course of pregnancy and childbirth and thus contributes to a significant decline in the number of cesarean sections and perinatal complications and a reduction of fetal and neonatal morbidity and mortality.

## Figures and Tables

**Table 1:** Maternal/fetal outcomes among body mass index (BMI) categories in the sample of pregnant women (*N* = 7101).

Maternal/Fetal Outcomes	Underweight *N* = 741 (10.5%)	Normal Weight *N* = 5400 (76.0%)	Overweight *N* = 602 (8.5%)	Obese *N* = 358 (5.0%)	*p*-Value
Prepregnancy BMI	17.7 ± 0.7	21.2 ± 1.6	26.8 ± 1.3	34.9 ± 3.7	<0.001
Maternal BMI before delivery	22.7 ± 1.8	26.3 ± 2.4	30.6 ± 2.5	37.2 ± 5.3	<0.001
Gestational weight gain	14.3 ± 4.9	14.0 ± 5.0	10.3 ± 6.8	6.5 ± 11.8	<0.001
C-section	211 (28.5)	1875 (34.7)	229 (38.0)	204 (57.0)	<0.001
Gestational hypertension	6 (0.8)	50 (0.9)	9 (1.5)	38 (10.6)	<0.001
Preeclampsia	8 (1.1)	56 (1.0)	17 (2.8)	39 (10.9)	<0.001
Gestational DM	18 (2.4)	100 (1.9)	25 (4.2)	31 (8.7)	<0.001
IUGR	13 (1.8)	65 (1.2)	7 (1.2)	14 (3.9)	<0.001
Smoking	15 (2.0)	84 (1.6)	14 (2.3)	26 (7.3)	<0.001
Low birth weight	43 (5.8)	262 (4.8)	36 (6.1)	8 (2.4)	n.s.
Macrosomia	35 (4.7)	378 (7.0)	55 (9.3)	32 (9.6)	<0.05

Data are mean +/− standard deviation or *n* (%); n.s.—nonsignificant.

**Table 2:** The relations among weight categories and selected maternal/fetal outcomes (multivariable logistic regression analysis).

Maternal/Fetal Outcomes	Underweight Adjusted OR (95% CI)	Normal Weight Adjusted OR (95% CI)	Overweight Adjusted OR (95% CI)	Obese Adjusted OR (95% CI)
C-section	0.8 (0.6–0.9) **	1	1.0 (0.9–1.2)	2.1 (1.7–2.8) ***
Gestational hypertension	0.9 (0.4–2.2)	1	1.5 (0.7–3.2)	15.3 (9.0–25.8) ***
Preeclampsia	1.1 (0.5–2.3)	1	3.4 (1.9–6.0) ***	13.2 (7.7–22.5) ***
Gestational DM	1.4 (0.9–2.4)	1	1.9 (1.2–2.9) **	2.4 (1.4–4.0) **
IUGR	1.3 (0.7–2.5)	1	0.6 (0.3–1.6)	3.7 (1.8–7.8) **
Low birth weight	1.1 (0.7–2.5)	1	0.9 (0.6–1.4)	0.2 (0.1–0.5) ***
Macrosomia	0.7 (0.5–0.9) *	1	1.7 (1.2–2.3) **	1.8 (1.2–2.7) **

OR—odds ratio; CI—confidence interval; * *p* < 0.05; ** *p* < 0.01; *** *p* < 0.001. Adjusted for maternal age, gestational age, gestational weight gain, and smoking.

**Table 3:** Maternal/fetal outcomes associated with GWG (IOM recommendations).

Maternal/Fetal Outcomes	GWG (IOM Recommendations)			*p*-Value
	Below	Range	Above	
	*N* = 2172 (31.9%)	*N* = 2738 (38.6%)	*N* = 2191 (30.9%)	
Weight gain (kg)	8.37 ± 2.69	13.50 ± 2.29	19.16 ± 4.20	<0.001
Prepregnancy BMI (kg/m^2^)	21.54 ± 3.38	21.67 ± 3.64	22.74 ±4.39	<0.001
Maternal BMI before delivery (kg/m^2^)	24.57 ± 2.96	26.53 ± 3.19	29.65 ± 4.01	<0.001
C-section (*N*)	744 (34.3)	932 (34.0)	843 (38.5)	<0.05
Gestational hypertension (*N*)	26 (1.2)	29 (1.1)	48 (2.2)	<0.05
Preeclampsia (*N*)	28 (1.3)	46 (1.7)	46 (2.1)	n.s.
Gestational DM (*N*)	63 (2.9)	67 (2.4)	44 (2.0)	n.s.
IUGR (*N*)	47 (2.2)	35 (1.3)	17 (0.8)	<0.001
Low birth weight (*N*)	197 (9.1)	95 (3.5)	57 (2.6)	<0.001
Macrosomia (*N*)	106 (4.9)	168 (6.1)	226 (10.3)	<0.001

Data are mean +/− standard deviation or *n* (%); GWG—gestational weight gain; n.s.—nonsignificant; *N* = number of cases.

**Table 4:** The relationships among GWG (IOM recommendations) and selected maternal/fetal outcomes (multiple logistic regression analysis).

Maternal/Fetal Outcomes	Inadequate GWG Adjusted OR (95% CI)	Excessive GWG Adjusted OR (95% CI)
C-section	0.9 (0.9–1.1)	1.2 (1.0–1.3) **
Gestational hypertension	1.1 (0.6–1.8)	1.7 (1.0–2.7) *
Preeclampsia	0.5 (0.3–0.9) *	0.9 (0.6–1.5)
Gestational DM	1.2 (0.9–1.8)	0.6 (0.4–0.9) *
IUGR	0.9 (0.6–1.5)	0.5 (0.3–1.0)
Low birth weight	2.0 (1.5–2.7) ***	0.9 (0.6–1.4)
Macrosomia	0.8 (0.6–1.1)	1.7 (1.3–2.1) ***

GWG—gestational weight gain; OR—odds ratio; CI—confidence interval; * *p* < 0.05; ** *p* < 0.01; *** *p* < 0.001. Adjusted for maternal age, gestational age, maternal BMI, and smoking.
